# Cytokine/Chemokine Expression Is Closely Associated Disease Severity of Human Adenovirus Infections in Immunocompetent Adults and Predicts Disease Progression

**DOI:** 10.3389/fimmu.2021.691879

**Published:** 2021-06-07

**Authors:** Jin Li, Jinli Wei, Zhixiang Xu, Chunmei Jiang, Mianhuan Li, Jie Chen, Yanjie Li, Minghui Yang, Yuchen Gu, Fuxiang Wang, Yuelong Shu, Yang Yang, Litao Sun, Yingxia Liu

**Affiliations:** ^1^ School of Public Health (Shenzhen), Sun Yat-sen University, Shenzhen, China; ^2^ Shenzhen Key Laboratory of Pathogen and Immunity, National Clinical Research Center for Infectious Disease, State Key Discipline of Infectious Disease, Shenzhen Third People’s Hospital, Second Hospital Affiliated to Southern University of Science and Technology, Shenzhen, China; ^3^ Department of Infectious Disease, The People’s Hospital of Longhua, Shenzhen, China; ^4^ Research and Development Department, Guangzhou Sagene Biotech Co., Ltd., Guangzhou, China

**Keywords:** human adenoviruses, cytokine storm, prediction, immunocompetent adults, disease progression

## Abstract

Increasing human Adenovirus (HAdV) infections complicated with acute respiratory distress syndrome (ARDS) even fatal outcome were reported in immunocompetent adolescent and adult patients. Here, we characterized the cytokine/chemokine expression profiles of immunocompetent patients complicated with ARDS during HAdV infection and identified biomarkers for disease severity/progression. Forty-eight cytokines/chemokines in the plasma samples from 19 HAdV-infected immunocompetent adolescent and adult patients (ten complicated with ARDS) were measured and analyzed in combination with clinical indices. Immunocompetent patients with ARDS caused by severe acute respiratory disease coronavirus (SARS-CoV)-2, 2009 pandemic H1N1 (panH1N1) or bacteria were included for comparative analyses. Similar indices of disease course/progression were found in immunocompetent patients with ARDS caused by HAdV, SARS-CoV-2 or panH1N infections, whereas the HAdV-infected group showed a higher prevalence of viremia, as well as increased levels of aspartate aminotransferase (AST), alanine aminotransferase (ALT) and creatine kinase (CK). Expression levels of 33 cytokines/chemokines were increased significantly in HAdV-infected patients with ARDS compared with that in healthy controls, and many of them were also significantly higher than those in SARS-CoV-2-infected and panH1N1-infected patients. Expression of interferon (IFN)-γ, interleukin (IL)-1β, hepatocyte growth factor (HGF), monokine induced by IFN-γ (MIG), IL-6, macrophage-colony stimulating factor (M-CSF), IL-10, IL-1α and IL-2Ra was significantly higher in HAdV-infected patients with ARDS than that in those without ARDS, and negatively associated with the ratio of the partial pressure of oxygen in arterial blood/fraction of inspired oxygen (PaO_2_/FiO_2_). Analyses of the receiver operating characteristic curve (ROC) showed that expression of IL-10, M-CSF, MIG, HGF, IL-1β, IFN-γ and IL-2Ra could predict the progression of HAdV infection, with the highest area under the curve (AUC) of 0.944 obtained for IL-10. Of note, the AUC value for the combination of IL-10, IFN-γ, and M-CSF reached 1. In conclusion, the “cytokine storm” occurred during HAdV infection in immunocompetent patients, and expression of IL-10, M-CSF, MIG, HGF, IL-1β, IFN-γ and IL-2Ra was closely associated with disease severity and could predict disease progression.

## Introduction

Human adenoviruses (HAdVs) are double-stranded, non-enveloped icosahedral viruses that typically cause mild infections involving the upper or lower respiratory tract, gastrointestinal tract, or conjunctiva (1–3). Until recently, a total of 104 subtypes within A–G species were identified, categorized either by virus neutralization assay or genomic/bioinformatics analyses (http://hadvwg.gmu.edu/). HAdV infections occurred mainly in children and immunocompromised patients, owing to a lack of humoral immunity or impaired immunity (1). In closed or crowded settings, HAdVs are highly contagious and epidemics may occur even in healthy children and adults (e.g., military recruits) (4–7). HAdV infections in the respiratory tract in immunocompetent adults can also occur, typically with mild and self-limiting symptoms in most cases (1, 2). As a result, HAdVs are rarely detected by clinicians and public-health surveillance in such settings. However, increasingly severe HAdV infections complicated by acute respiratory distress syndrome (ARDS) in immunocompetent adolescents and adults have been reported with a mortality prevalence of ≤80% (8–16), which poses a serious threat to public health. The ongoing global pandemic due to severe acute respiratory coronavirus-2 (SARS-CoV-2) infection and seasonally prevalent 2009 pandemic H1N1 (panH1N1) infection can cause severe infections in immunocompetent adults (17–20). Due to distinct prognoses and treatments among these respiratory viruses, differentiating HAdV infections accurately and in a timely manner is crucial for clinicians and epidemiologists.

The “cytokine storm” (CS) is the release of circulating cytokines/chemokines during an acute infection. The CS has been shown to be correlated directly with acute lung injury and the development of ARDS during viral infections (21, 22), including influenza viruses and coronaviruses (23–30). Moreover, some cytokines/chemokines have been found to predict the disease progression accurately (24, 26, 31–33). HAdV has also been shown to be a proinflammatory virus that can trigger the release of high levels of inflammatory cytokines and chemokines in children patients, and the expression levels differed among different disease severity (34–36). However, the specific expression profiles of cytokines/chemokines in immunocompetent adolescent and adult patients, especially those complicated with ARDS caused by HAdV infection, have rarely been reported.

In this study, we characterized the cytokine/chemokine expression profiles of immunocompetent patients complicated with ARDS during HAdV infection, and compared them against other common causative agents of ARDS, including SARS-CoV-2, panH1N1 and bacteria. Furthermore, we identified biomarkers for disease severity and progression in HAdV infections.

## Materials and Methods

### Ethical Approval of the Study Protocol

The study protocol was approved (SZTHEC2016001) by the Ethics Committees of Shenzhen Third People’s Hospital (Shenzen, China). Written informed consent was obtained from patients infected with HAdV, panH1N1, or suffering from bacterial pneumonia. Verbal informed consent was obtained from coronavirus 2019 (COVID-19) patients because pen and paper were not allowed in containment facilities. The study was conducted in accordance with the International Conference on Harmonization Guidelines for Good Clinical Practice, the Declaration of Helsinki 1965 and later amendments, and the ethical guidelines of Shenzhen Third People’s Hospital.

### Patient Information and Data Collection

This was a retrospective study. Study participants were immunocompetent patients hospitalized in Shenzhen Third People’s Hospital from 2018 to 2020. Nineteen laboratory analyses-confirmed HAdV-infected patients (10 complicated with ARDS and nine without ARDS) were enrolled. Patients complicated with ARDS caused by infection with SARS-CoV-2 (n = 28), panH1N1 (n = 21), suffering from bacterial pneumonia (n = 10) and healthy controls (HCs) (n = 8) were also included for analyses. All patients complicated with ARDS met the criteria of the Berlin definition for the diagnosis of ARDS (37). Plasma and respiratory specimens were collected from patients and HCs. Clinical information, including complete blood counts and blood biochemistry, were collected at the earliest time point after hospitalization. Immunocompromised patients infected with the human immunodeficiency virus, suffering from neutropenia, receiving immunosuppressive chemotherapy, with malignant neoplasms, or pregnant or breastfeeding women, were excluded, as reported previously (38).

### Real-Time Reverse Transcription-Quantitative Polymerase Chain Reaction (RT-qPCR) and Next-Generation Sequencing (NGS)

Sputum or nasopharyngeal swabs were collected from patients at various time-points after hospitalization. RT-qPCR was undertaken as described previously (39), and using commercial qRT-PCR kits for the detection of HAdV (Mabsky Biotech Co., Ltd., Shenzhen, China). Samples positive for HAdVs were subjected to virus isolation using HEP-2 cells and NGS (Guangzhou Sagene Biotech, Guangzhou, China).

### Measurement of Expression of Cytokines and Chemokines

The plasma samples of laboratory analyses-confirmed patients were collected at the earliest possible time-point after hospitalization and thereafter. The plasma of HCs was included as the negative control. The concentrations of 48 cytokines including adaptive immunity cytokines, pro-inflammatory cytokines, and anti-inflammatory cytokines associated with infectious diseases and inflammation were measured using the Bio-Plex Pro Human Cytokine Screening Panel (Bio-Rad Laboratories, Hercules, CA, USA) on a Luminex™ 200 instrument (Merck Millipore, Burlington, MA, USA) following manufacturer instructions, as reported previously (31).

### Quantification of Hypoxia and Lung Injury

The partial pressure of oxygen in arterial blood (PaO_2_) was measured by a blood-gas analyzer (ABL90; Radiometer, Copenhagen, Denmark) at various time-points after hospitalization, as reported previously (27, 31). The fraction of inspired oxygen (FiO_2_) was calculated using the following formula: FiO_2_ = (21 + oxygen flow [in units of L/minute] × 4)/100. The PaO_2_/FiO_2_ ratio (in mmHg) was obtained by dividing the PaO_2_ value with the FiO_2_ value. A PaO_2_/FiO_2_ ratio ≤100 mmHg is considered one of the criteria for severe ARDS, as defined previously (37).

### Statistical Analysis

The Mann–Whitney *U*-test was employed to determine the differences between groups of continuous variables. The Fisher exact test was used for categorical variables. The Spearman correlation coefficient was employed to analyze the linear correlation. The area under the receiver operating characteristic (ROC) curve (AUC) of plasma cytokine levels was estimated for patients developing ARDS or not developing ARDS. Moreover, the combined values for the prediction of developing ARDS was calculated using binary logistic regression. Statistical values were calculated using SPSS 20.0 (IBM, Armonk, NY, USA). A P-value of 0.01–0.05, 0.001–0.01 and <0.001 was considered significant, very significant, and extremely significant, respectively.

## Results

### Distinct Clinical Characteristics of Immunocompetent Patients Complicated With ARDS Infected by HAdV, SARS-CoV-2, panH1N1 or Bacteria

Nineteen patients with laboratory analyses-confirmed HAdVs with ten patients complicated by ARDS (case No. 01-10) were included in this study, The patients were predominantly male (78.9%) and <60 years of age (94.7%) (**Table S1**). Patients initially showed influenza-like symptoms that developed rapidly into pneumonia and ARDS within 6 days after illness onset (d.a.o) in most cases of the ARDS group (**Table S1**). Moreover, only HAdV-7 (8/10) and HAdV-55 (2/10) were found in the ARDS group, and seven patients received antiviral treatment with cidofovir (**Table S1**). To compare the distinct characteristics of HAdV infections with those of other important respiratory infections, clinical information was first collected and analyzed from immunocompetent patients with ARDS caused by SARS-CoV-2 (n = 28, collected in 2020), panH1N1 (n = 21, collected during 2018–2019) and bacteria (n = 10, collected in 2019) (**Table S2**). The median age of the HAdV-infected group was significantly younger than that of the other groups, and also a lower instance of underlying diseases. The median number of days from disease onset to hospital admission and hospitalization duration were similar among the four groups. There was no significant difference in the proportion of patients developing severe ARDS, needing mechanical ventilation or transfer to the intensive unit care unit (ICU), or in-hospital prevalence of death (**Table S2**). However, compared with patients infected with SARS-CoV-2 or panH1N1, a higher prevalence of hepatic insufficiency and cardiac failure was found in HAdV-infected patients, though the difference was not significant in some cases. Notably, a significantly higher prevalence of viremia in HAdV-infected patients (90%) was found (**Table S2**). The prevalence of increased levels of aspartate aminotransferase (AST), alanine aminotransferase (ALT) and creatine kinase (CK) in HAdV-infected patients upon hospital admission was significantly higher than that in the other groups, especially compared with SARS-CoV-2-infected and panH1N1-infected patients (**Table 1**). Moreover, the prevalence of lymphopenia in HAdV-infected patients was significantly higher than that in SARS-CoV-2-infected cases and bacterial-pneumonia patients (**Table 1**). Also, the prevalence of leukopenia and thrombocytopenia in HAdV-infected patients was significantly higher than that in panH1N1-infected patients (**Table 1**).

**Table 1:** Laboratory results of hospitalized patients complicated with ARDS caused by HAdV, SARS-CoV-2, panH1N1 and bacteria.

Parameter ^a^	HAdV^c^	SARS-CoV-2	P value	panH1N1	P value	Bacteria	P value
WBC (× 10^9^/L) ^b^	5.12 (3.62, 7.33)	4.57 (3.35, 5.82)	0.241	8.66 (5.42, 14.04)	0.010	14.62 (7.96, 23.93)	0.043
LYM (× 10^9^/L) ^b^	0.77 (0.46, 0.86)	1.14 (0.97, 1.42)	0.000	0.82 (0.52, 1.16)	0.347	1.06 (0.39, 1.63)	0.240
LYM (%) ^b^	15 (9.3, 19.3)	21.35 (14.7, 26.68)	0.085	8.7 (4.5, 15.5)	0.250	7.3 (4.9, 10.53)	0.371
NEU (× 10^9^/L) ^b^	5.72 (3.16, 6.81)	2.89 (1.61, 3.64)	0.004	6.93 (4.28, 12.36)	0.079	13.06 (4.87, 21.99)	0.123
NEU (%) ^b^	83.2 (75.9, 83.9)	68.55 (60.5, 78.43)	0.016	84.2 (80.7, 88.5)	0.369	87 (75.23, 91.63)	0.594
PLT (× 10^9^/L) ^b^	128.5 (119, 191.5)	173 (140.5, 190)	0.191	180 (156, 218)	0.085	181.5 (85, 275)	0.494
AST (U/L) ^b^	123.15 (89.25, 244)	29.5 (22, 42.25)	<0.001	35.1 (28.6, 57.1)	<0.001	149.05 (34.25, 324.1)	0.796
ALT (U/L) ^b^	79.5 (55.9, 111.38)	24.5 (17.25, 27)	<0.001	47.9 (34.4, 75.4)	0.028	51.5 (28.18, 80.6)	0.218
TB (umol/L)	11.9 (7.5, 15.6)	9.4 (8.1, 11.95)	0.652	11.9 (8.1, 17)	0.765	12.05 (10.73, 23.63)	0.679
CRE (µmol/L) ^b^	84.6 (70.5, 91.8)	73.35 (56.5, 81.25)	0.216	69.55 (44.1, 97.8)	0.165	117.5 (82, 363.55)	0.321
BUN ^b^	3.23 (2.87, 3.39)	5.05 (3.77, 5.77)	0.002	5.91 (3.49, 7.32)	0.059	9.39 (5.78, 16.53)	0.030
CK, MB ^b^	1.09 (0.94, 1.38)	0.73 (0.34, 1.01)	0.226	1.55 (0.96, 5.44)	0.620	6.54 (1.65, 11.48)	0.109
CK (U/L) ^b^	748 (574.7, 827.7)	67 (53, 126)	0.001	198.6 (127.5, 344.9)	0.001	719 (316, 2140.8)	0.662
CRP (nmol/L) ^b^	111 (84.91, 136.69)	14.05 (6.53, 29.27)	<0.001	111.2 (40.4, 265.1)	0.886	228.01 (170.3, 261.1)	0.016
ALB (g/L) ^b^	29.8 (28.35, 32.28)	38.8 (37.45, 41.95)	0.001	33.7 (30.4, 35.48)	0.133	31.3 (27.7, 34.98)	1.000
LDH (U/L) ^b^	1279 (467, 4535)	302 (180.25, 477)	0.025	757 (403, 1109)	0.319	519.5 (310.3, 746.75)	0.524
PCT (ng/mL) ^b^	1.74 (1.11, 3.23)	0.05 (0.03, 0.08)	0.000	0.79 (0.22, 2.32)	0.356	29.59 (0.626, 100)	0.350
CD4 (count/μl) ^b^	216.5 (151.5, 271.3)	336.5 (208, 456.25)	0.091	202 (124.25, 436.75)	0.721	195.5 (148.5, 417.25)	1.000
CD8 (count/μl) ^b^	178 (86.75, 288.25)	146 (118.25, 232)	0.860	135 (82, 181.75)	0.281	122.5 (35.75, 195)	0.352
Leukopenia	5/10 (50%)	10/28 (35.7%)	0.473	0/21 (0%)	0. 002	2/10 (20%)	0.350
Lymphopenia	10/10 (100%)	8/28 (28.6%)	<0.001	16/21 (76.2%)	0.147	5/10 (50%)	0.033
Neutropenia	0/10 (0%)	2/28 (7.1%)	1.000	0/21 (0%)	1.000	1/10 (10%)	1.000
Neutrophilia	1/10 (10%)	0/28 (0%)	0.263	9/21 (42.9%)	0.106	7/10 (70%)	0.020
Thrombocytopenia	7/10 (70%)	9/28 (32.1%)	0.062	3/21 (14.3%)	0.004	4/10 (40%)	0.370
Hypoalbuminemia	6/8(75%)	3/28(10.7%)	0.001	8/20(40%)	0.208	5/10(50%)	0.367
Elevated AST	10/10 (100%)	3/28 (10.7%)	<0.001	9/21 (42.9%)	0.004	6/10 (60%)	0.087
Elevated ALT	10/10 (100%)	0/28 (0%)	<0.001	5/21 (23.8%)	<0.001	6/10 (60%)	0.087
Elevated CRE	1/9 (11.1%)	2/28 (7.1%)	1.000	5/21 (23.8%)	0.637	5/8 (62.5%)	0.050
Elevated CK	6/6 (100%)	1/19 (5.3%)	<0.001	9/19 (47.4%)	0.051	4/7 (57.1%)	0.192
Elevated CRP	9/9 (100%)	16/28 (57.1%)	0.018	20/21 (95.2%)	1.000	10/10 (100%)	1.000
Elevated LDH	4/5 (80%)	16/28 (53.6%)	0.625	12/17 (70.6%)	1.000	7/8 (87.5%)	1.000

a Results were obtained from patients at the earliest available time-point after hospitalization.

b Values shown represent the mean and inter-quartile range (IQR).

c Reference group.

### Comparison of Cytokines/Chemokines Expression Profiles Amongst Immunocompetent Patients Complicated With ARDS Infected by HAdV, SARS-CoV-2, panH1N1 or Bacteria

**Figure 1:**
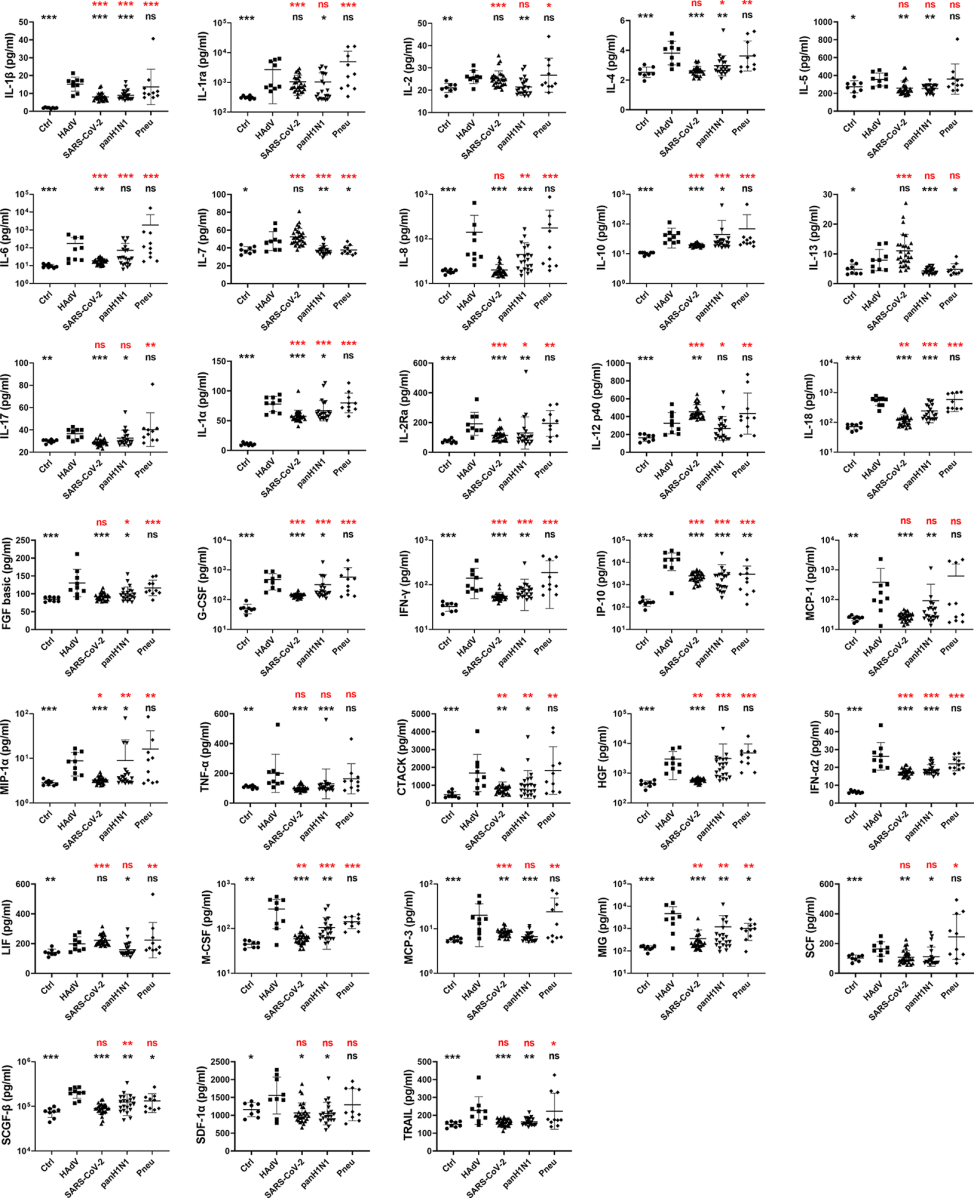
Comparison of cytokines/chemokines expression profiles among patients infected with HAdV, SARS-CoV-2, panH1N1 or bacteria complicated with ARDS upon admission. Samples from patients infected with HAdV (n = 9), SARS-CoV-2 (n = 28) and panH1N1 (n = 21) were collected at the earliest possible time-point after hospitalization and subjected to cytokines/chemokines measurement. Healthy controls (n = 8) and patients with bacterial pneumonia (n = 10) were included as controls. Results of 33 cytokines/chemokines showing significantly increased expression in the HAdV group are shown. Statistical analyses with the reference group of HAdV and HCs are shown in black and red, respectively. P = 0.01–0.05, 0.001–0.01 and <0.001 was considered significant (*), very significant (**) and extremely significant (***), respectively, whereas ns represents not significant.

Expression levels of cytokines/chemokines indicate disease severity in some other respiratory infections (24, 26, 27, 31). Hence, the concentrations of 48 cytokines/chemokines including adaptive immunity cytokines, pro-inflammatory cytokines, and anti-inflammatory cytokines associated with infectious diseases and inflammation were measured and compared in patients infected with HAdV, SARS-CoV-2, panH1N1, or suffering from bacterial pneumonia complicated with ARDS (**Figures 1** and **S1**). Expression of 33 cytokines/chemokines was significantly higher in HAdV-infected patients complicated with ARDS than that in HCs (**Figures 1** and **S1**), and expression levels of most of these cytokines were significantly positively correlated with the HAdV load (**Figure S2**). Among these 33 cytokines/chemokines, significantly increased expression of interleukin (IL)-1α, IL-1β, IL-2Ra, IL-10, IL-18, granulocyte-colony stimulating factor (G-CSF), interferon (IFN)-α2, IFN-γ, interferon gamma-induced protein (IP)-10, macrophage inflammatory protein (MIP)-1α, cutaneous T cell-attracting chemokine (CTACK), macrophage-colony stimulating factor (M-CSF), and monokine induced by IFN-γ (MIG) were also found in the remaining three groups, whereas concentrations in the HAdV-infected group were significantly higher than those in the SARS-CoV-2-infected group and panH1N1-infected group (**Figures 1** and **S1**). Interestingly, significantly increased expression of monocyte chemoattractant protein (MCP)-1, tumor necrosis factor (TNF)-α and stromal cell-derived factor (SDF)-1α was found only in HAdV-infected patients among the four groups, and significantly increased levels of IL-17, Skp, Cullin, F-box containing complex (SCF)-β and tumor necrosis factor-related apoptosis inducing ligand (TRAIL) were found specifically in the HAdV-infected group among the three viral infection groups (**Figures 1** and **S1**). These results indicated that the CS occurred in HAdV infection, and that it was possibly even more severe than that in SARS-CoV-2 and panH1N1 infections in immunocompetent patients.

### Differential Expression of Cytokines/Chemokines in HAdV Patients Complicated With or Not Complicated With ARDS

**Figure 2:**
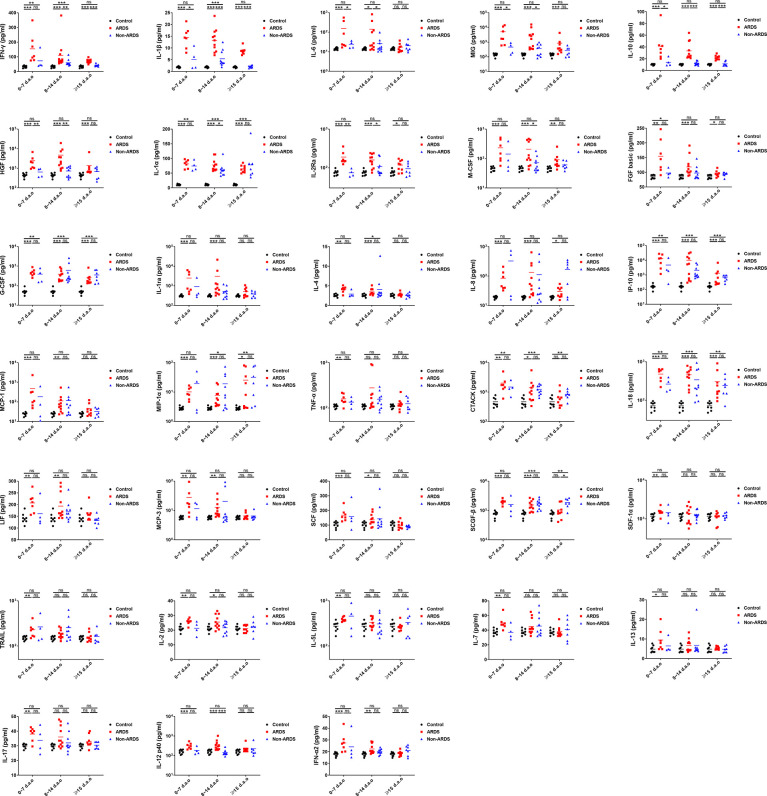
Comparison of cytokines/chemokines showing significant increases in HAdV patients with or without ARDS during disease progression. Expression of 33 cytokines/chemokines measured at different days after illness onset were compared between HAdV patients with or without ARDS. P = 0.01–0.05, 0.001–0.01 and 0.0001–0.001 was considered significant (*), very significant (**) and extremely significant (***), respectively, whereas ns represents not significant.

We wished to investigate the cytokines/chemokines that might associated with the disease severity of HAdV infection. Hence, the expression profile of these 33 cytokines/chemokines in patients with or without ARDS at different phases of disease were analyzed further (**Figure 2**). The date of sample collection was stratified into three groups according to disease progression, as reported previously (32), the first 7 days after illness onset (0~7 d.a.o), between 8 and 14 days following illness onset (8~14 d.a.o), and during the recovery phase from 15 days after disease onset (≥ 15 d.a.o). During the first 7 d.a.o, expression of all 33 cytokines/chemokines was increased in HAdV-infected patients with ARDS, whereas expression of only IFN-γ, IL-12, IL-18, fibroblast growth factor (FGF) basic, G-CSF, IP-10 and CTACK was increased in patients not suffering from ARDS. During 8~14 d.a.o, expression of TNF-α, SDF-1α, TRAIL, IL-5, IL-7, IL-13 and IL-17 in patients with ARDS returned to normal, and expression of 10 cytokines/chemokines (IFN-γ, IL-1α, IL-1β, IL-18, IL-4, MIP-1α, G-CSF, IP-10, CTACK and SCF-β) was increased in patients without ARDS. Furthermore, expression of nine cytokines/chemokines (hepatocyte growth factor (HGF), MIG, IFN-γ, IL-1β, IL-6, M-CSF, IL-10, IL-1α and IL-2Ra) was significantly higher in HAdV-infected patients with ARDS than in those without ARDS during disease progression. Moreover, higher expression of MIG, M-CSF, IL-6, IL-1α and IL-2Ra was mainly found within 14 d.a.o, and higher expression of IFN-γ, IL-1β and IL-10 was found during the entire disease progression. Notably, most of these cytokines/chemokines showing significantly increased expression in patients with ARDS showed an obvious trend of decrease after 14 d.a.o. These results suggested a possible role of cytokines/chemokines in the pathogenesis of HAdV infection, especially the differentially expressed cytokines/chemokines between patients with or without ARDS.

### Expression of IL-10, M-CSF, MIG, HGF, IL-1β, IFN-γ and IL-2Ra Was Closely Associated With Disease Severity and Could Predict Disease Progression in HAdV Infection

**Figure 3:**
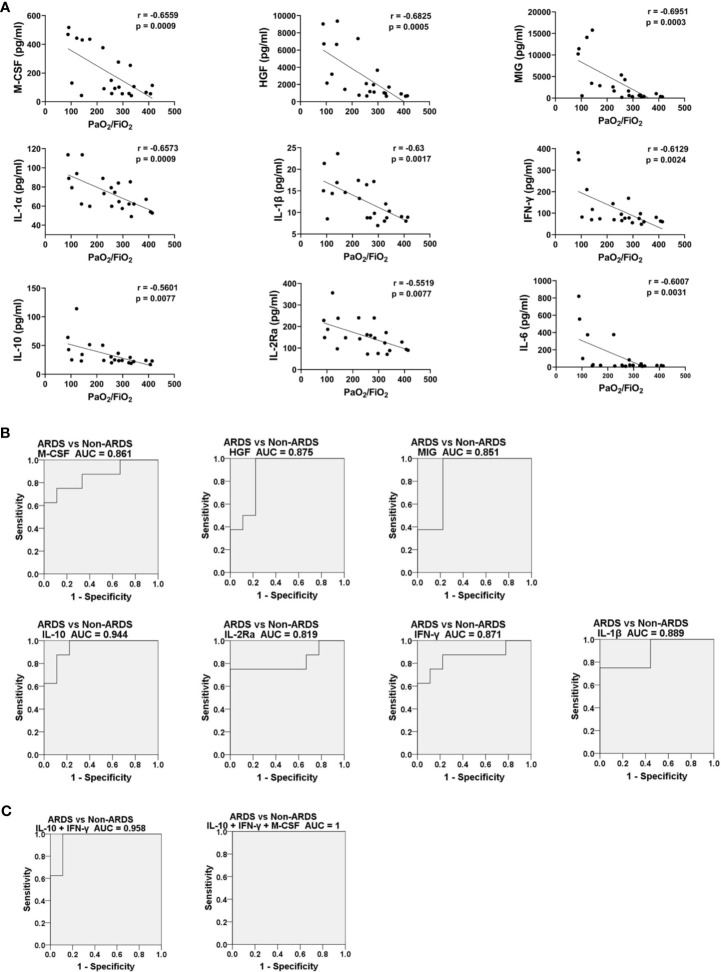
Expression of IL-10, M-CSF, MIG, HGF, IL-1β, IFN-γ and IL-2Ra was closely associated with disease severity and could predict disease progression. **(A)** Spearman rank correlation analysis between expression of HGF, MIG, IFN-γ, IL-1β, IL-6, M-CSF, IL-10, IL-1α and IL-2Ra measured from plasma samples and the corresponding PaO_2_/FiO_2_ ratio on the same day. **(B)** ROC curves of expression levels of HGF, MIG, IFN-γ, IL-1β, M-CSF, IL-10, and IL-2Ra upon hospital admission for HAdV-infected patients with and without ARDS during hospitalization. **(C)** ROC curves of different combination of IL-10, IFN-γ, and M-CSF expression levels upon hospital admission for HAdV-infected patients with and without ARDS during hospitalization. All P-values for the ROC curves were <0.05.

It has been shown that concentrations of specific cytokines/chemokines are associated with disease severity in H7N9, H5N1, H5N6 and SARS-CoV-2 infections (24, 26, 27, 31, 40, 41). Spearman coefficient correlation analysis was done to analyze the association between expression of the nine differentially expressed cytokines/chemokines and the PaO_2_/FiO_2_ ratio in HAdV-infected patients (**Figure 3A**). The concentration of the nine cytokines/chemokines was negatively correlated with the PaO_2_/FiO_2_ ratio, with the *r* values ranging from −0.5519 (IL-2Ra) to −0.6915 (MIG) and P < 0.05, which indicated that these cytokines/chemokines were associated with disease severity in HAdV infection. The dynamic change in expression of these nine cytokines/chemokines in HAdV-infected patients complicated with ARDS was also measured and analyzed (**Figure S3**). The concentrations of these cytokines/chemokines in most cases showed an obvious decreasing trend during disease progression. In addition, we calculated the AUC to test whether the nine cytokines/chemokines could be biomarkers for the prediction of developing ARDS in HAdV-infected patients. These 17 HAdV-infected patients were divided into an ARDS group and non-ARDS group. Expression of the nine cytokines/chemokines at the earliest time-point after hospital admission was used for the calculation (**Figures 3B** and **S4A**). The AUC for IL-10, M-CSF, MIG, HGF, IL-1β, IFN-γ and IL-2Ra was >0.8 and P < 0.05, and IL-10 possessed the highest AUC (0.944). Then we further analyzed the AUC values of different combination of IL-10 with the other ones (**Figure 3C**, **Figure S4B, C**), and found that combination of IL-10 and M-CSF increased the value of AUC to 0.958 (**Figure 3C**). More surprisingly, the AUC value for the combination of IL-10, IFN-γ, and M-CSF reached 1 (**Figure 3C**).

## Discussion

Initially, most respiratory infections show influenza-like symptoms. ARDS (which carries high mortality and morbidity) serves as a common complication during severe respiratory infections (37). Compared with severe infection with SARS-CoV-2 or panH1N1, the overall disease course of severe HAdV infection (including the interval from disease onset to hospital admission and hospitalization duration) and disease progression (including developing severe ARDS, utilization of mechanical ventilation, requiring ICU transfer, and the in-hospital mortality prevalence) were similar (**Table S2**). These data indicated that the severity of ARDS caused by these three viruses was similar. However, unlike influenza viruses and SARS-CoV-2, laboratory testing of HAdVs is not a routine examination for patients with pneumonia in most hospitals. Thus, it is easy to ignore HAdV-induced severe infections, especially for immunocompetent adolescents and adults (42). Apart from the laboratory-based diagnosis, distinct clinical characteristics could also provide some hints for clinicians. For example, severe infection with SARS-CoV-2 or panH1N1 was found mainly in elderly patients with underlying diseases, whereas most HAdV-infected patients with ARDS reported in this study and previous studies were under 60 years of age (12, 43, 44), much younger than those infected with SARS-CoV-2 or panH1N1 (**Table S2**). Meanwhile, with regard to complete blood counts and blood biochemistry, severe HAdV infections were characterized by significantly higher levels of AST, ALT, and CK than those in SARS-CoV-2-infected and panH1N1-infected patients (**Table 1**). Therefore, immunocompetent adolescents and young adults with respiratory symptoms and significantly elevated AST, ALT, and CK were proposed to be at higher risk of HAdV infection when compared with other respiratory viruses. Currently, options for efficacious antiviral treatment of HAdV infections are limited. Studies have shown that cidofovir and its lipid ester analog, brincidofovir, may be the most promising drugs (2, 45). Fortunately, HAdV infections were confirmed by laboratory analyses within 5 days after hospital admission for all patients with ARDS, and six patients received cidofovir treatment and survived in our study cohort (**Table S1**). These results highlight the importance of a timely laboratory-based diagnosis of HAdV infection and the benefit of cidofovir treatment for severe HAdV infection. Similar to data from other studies (2, 46), HAdV-7 and HAdV-55 in species B were the main serotypes that caused severe pneumonia in immunocompetent adolescent and adults, and the underlying mechanism merits further investigation.

The CS with uncontrolled proinflammatory responses has important roles in the immunopathogenesis of infection and is associated with the severity and outcome of disease (21, 22, 47, 48). Increased expression of cytokines/chemokines, and several clinical abnormalities reflecting hyperinflammation and tissue damage in CS disorders (22), (e.g., increased levels of C-reactive protein and lactate dehydrogenase, hypoalbuminemia, thrombocytopenia, hepatic and renal insufficiency) were also observed in HAdV-infected patients (**Table 1**), indicating that the CS occurred following HAdV infection. Totally, 33 tested cytokines/chemokines were found to be significantly elevated in HAdV patients with ARDS, among which elevation of IP-10, TNF-α, IL-1α, IL-1β, IL-6, MIP-1α, MIG, and IFN-γ have also been found from children patients in previous studies (34, 49). When comparing with SARS-CoV-2 or panH1N1 infections, some cytokines were commonly elevated, while elevated cytokines including MCP-1, TNF-α, SDF-1α, IL-17, SCF-β, and TRAIL were found specifically in the HAdV-infected group (**Figure 1** and **Figure S1**). Moreover, many of the cytokines/chemokines showing increased expression in HAdV-infected patients complicated with ARDS also showed significantly higher expression than those with SARS-CoV-2 or panH1N1 infections (**Figures 1** and **S1**). These results suggested the common and specific roles of some cytokines in different respiratory infections and possibly a more severe CS in HAdV-infected patients complicated with ARDS than SARS-CoV-2 or panH1N1, which merits further investigation. Furthermore, the concentrations of nine cytokines including IL-6, M-CSF, IL-1β, MIG which are monocyte/macrophage activation associated biomarkers (41), IFN-γ which is the sole type II IFN and important in early host defense against infection (50), IL-1α which activates the inflammatory process (51), IL-10 which regulates and suppresses the expression of proinflammatory cytokines (52), HGF, and IL-2Ra were significantly higher in HAdV-infected patients complicated with ARDS, and negatively correlated with the PaO_2_/FiO_2_ ratio (**Figures 2**, **3**). Hence, these cytokines might have crucial roles in the pathophysiology of HAdV infection. Moreover, studies have aimed to establish prediction models for disease progression based on expression of cytokines/chemokines in plasma (24, 26, 31–33). Specific cytokines/chemokines could accurately predict disease progression, such as IP-10 for COVID-19, and Ang II for H7N9 infection (24, 26, 31–33). Unlike the other viruses, IL-10 in HAdV infection serves as the best predictor for disease progression with the highest area under the curve (AUC) of 0.944 (**Figure 3B**), indicating that predictors for different viral infections were virus specific. Notably, we also found the AUC value for the combination of IL-10, IFN-γ, and M-CSF reached 1 (**Figure 3C**), therefore, expression levels the three cytokines might fully predict the disease severity of HAdV infection. Given the crucial role of the CS in severe inflammation and damage to vital organs, short courses of treatment with corticosteroids at low-to-moderate doses might be beneficial against severe infections by HAdVs, panH1N1, H7N9 and SARS-CoV-2 (53, 54).

In summary, the CS occurred during HAdV infection. Expression of IL-10, M-CSF, MIG, HGF, IL-1β, IFN-γ and IL-2Ra was closely associated with disease severity and could predict disease progression. These results aid understanding of the clinical characteristics and immunopathologic mechanisms of severe HAdV infection in immunocompetent adolescents and adults. Our data also highlight the increasing public-health threat of emerging (or re-emerging) HAdV subtypes, especially HAdV-7 and HAdV-55.

## Age Specific Reference Ranges Used to Define Abnormalities in Blood Results

Leukopenia (× 10^9^/L): 2 months-2 years: <5, >2 years: <4; Lymphopenia (× 10^9^/L): 2-11 months: <4.0, 1-11 years: <1.5, 12+ years: <1; Neutropenia (× 10^9^/L): All ages: <1.5; Neutrophilia (× 10^9^/L): All ages: >8.5; Thrombocytopenia (× 10^9^/L): All ages: <150; Elevated AST(U/L): All ages: >50; Elevated ALT (U/L): All ages: >50; Elevated CRE (µmol/L): All ages: >120; Elevated CK (U/L): All ages: >200; Elevated CRP (nmol/L): All ages: >10; Elevated LDH (U/L): <7 years: >400, 7-15 years: >300, 16+ years: >250.

## Data Availability Statement

The original contributions presented in the study are included in the article/**Supplementary Material**. Further inquiries can be directed to the corresponding author.

## Ethics Statement

The studies involving human participants were reviewed and approved by Ethics Committees of Shenzhen Third People’s Hospital. Written informed consent to participate in this study was provided by the participants’ legal guardian/next of kin.

## Author Contributions

JL, YY, and JC contributed to the analysis and interpretation of data. JW, ZX, ML, MY, CJ, YJL, YG, and FW enrolled the patients and collected the data. YS, LS, and YXL were responsible for the concept and design of the study; JL drafted the article. YXL, LS, YS, and YY were responsible for the critical revision for important intellectual content and for the final approval of the article. All authors agree to be accountable for the content of the work. All authors contributed to the article and approved the submitted version.

## Funding

This work was supported by grants from the Shenzhen Science and Technology Research and Development Project [grant no. 202002073000001 and JSGG20200225152008136], National Natural Science Foundation of China [grant no. 31971147] and Shenzhen Science and Technology Innovation Commission [grant no. JCYJ20190807155011406 and KQTD20180411143323605] and National Science and Technology Major Project [grant no. 2018ZX10713001], the Ministry of Science and Technology (2020YFC0846300).

## Conflict of Interest

JC was employed by the company Guangzhou Sagene Biotech.

The remaining authors declare that the research was conducted in the absence of any commercial or financial relationships that could be construed as a potential conflict of interest.

## Supplementary Material

Supplementary Figure 1 HeatMap of differentially expressed cytokines/chemokines. The HeatMap of the differentially expressed cytokines/chemokines between HAdV-infected patients against **(A)** healthy controls, **(B)** SARS-CoV-2-infected patients, **(C)** panH1N1-infected patients **(C, D)** bacterial-pneumonia patients.

Supplementary Figure 2 Correlation between cytokines/chemokines expression and HAdV viral load. Expression of the 26 cytokines/chemokines showing significant increases measured from plasma samples collected upon hospital admission and thereafter and the corresponding viral load (indicated as log (copies/mL)) on the same day were evaluated using Spearman rank correlation analysis.

Supplementary Figure 3 Dynamic changes in expression of nine differentially expressed cytokines/chemokines measured in HAdV patients with or without ARDS at the indicated time-points from HAdV patients complicated with ARDS. The fatal case (case 06) is marked in red.

Supplementary Figure 4
**(A)** ROC curves of expression levels of IL-6 and IL-1α. **(B)** ROC curves of the combination of IL-10 with HGF, MIG, IL-1β, M-CSF, IL-10, and IL-2Ra expression levels upon hospital admission for HAdV-infected patients with and without ARDS during hospitalization. **(C)** ROC curves of the combination of IL-10, IFN-γ with HGF, MIG, IL-1β, and IL-2Ra expression levels upon hospital admission for HAdV-infected patients with and without ARDS during hospitalization. All P-values for the ROC curves were <0.05 except IL-6 and IL-1α.

## Abbreviations

WBC, white blood cell; LYM, lymphocyte; NEU, neutrophil; PLT, platelet; AST, aspartate aminotransferase; ALT, alanine aminotransferase; CRE, serum creatinine; BUN, blood urea nitrogen; CK, creatine kinase; CRP, C-reactive protein; LDH, lactate dehydrogenase.
