# Supplementary figures and images for: Cytokine/Chemokine Expression Is Closely Associated Disease Severity of Human Adenovirus Infections in Immunocompetent Adults and Predicts Disease Progression
Source: Front Immunol. 2021 Jun 7;12:691879. doi: 10.3389/fimmu.2021.691879 (PMC8215364; doi:10.3389/fimmu.2021.691879)

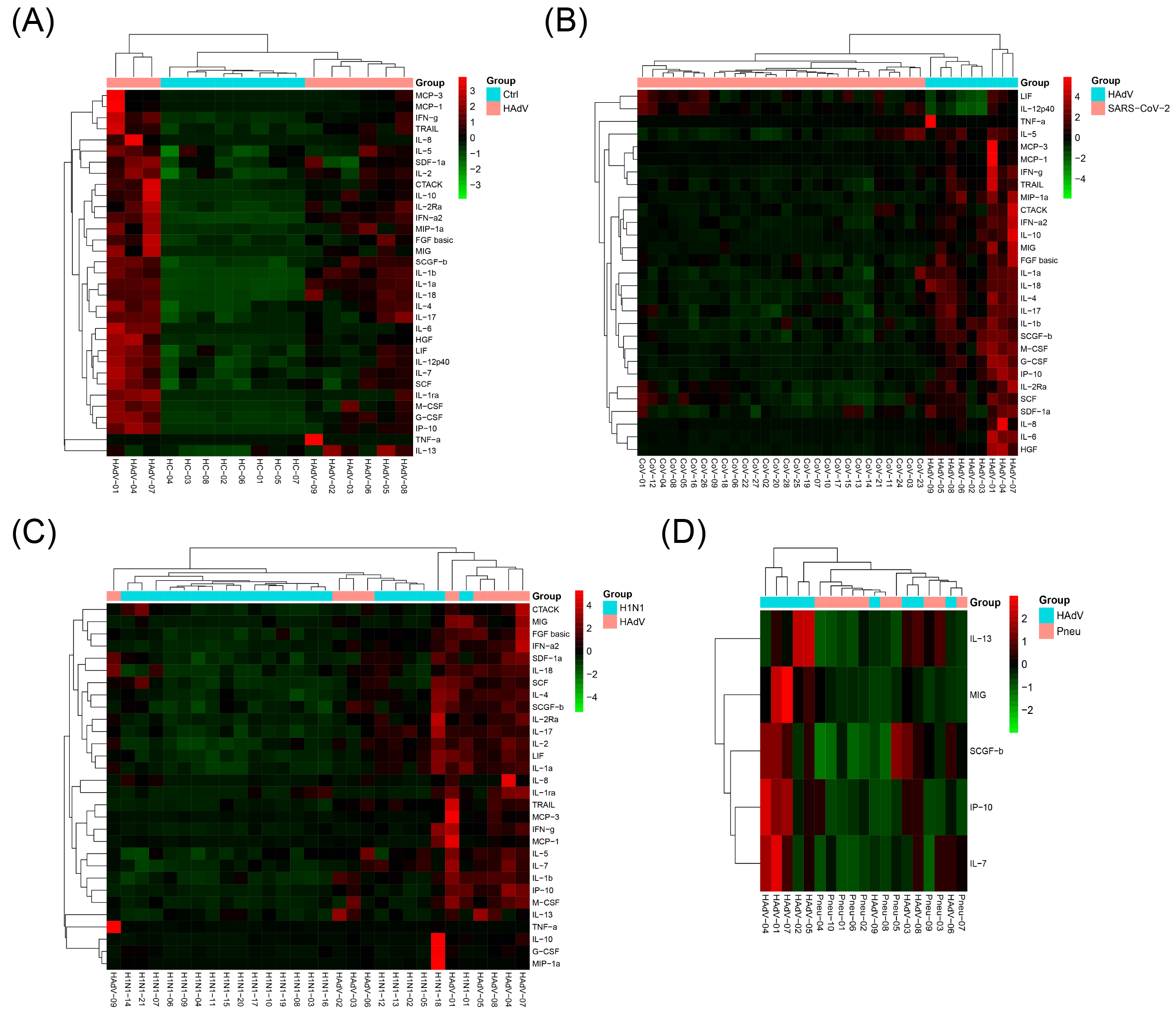

Supplement: Supplementary Figure 1 — HeatMap of differentially expressed cytokines/chemokines. The HeatMap of the differentially expressed cytokines/chemokines between HAdV-infected patients against (A) healthy controls, (B) SARS-CoV-2-infected patients, (C) panH1N1-infected patients (C, D) bacterial-pneumonia patients. [file Image_1.tif]

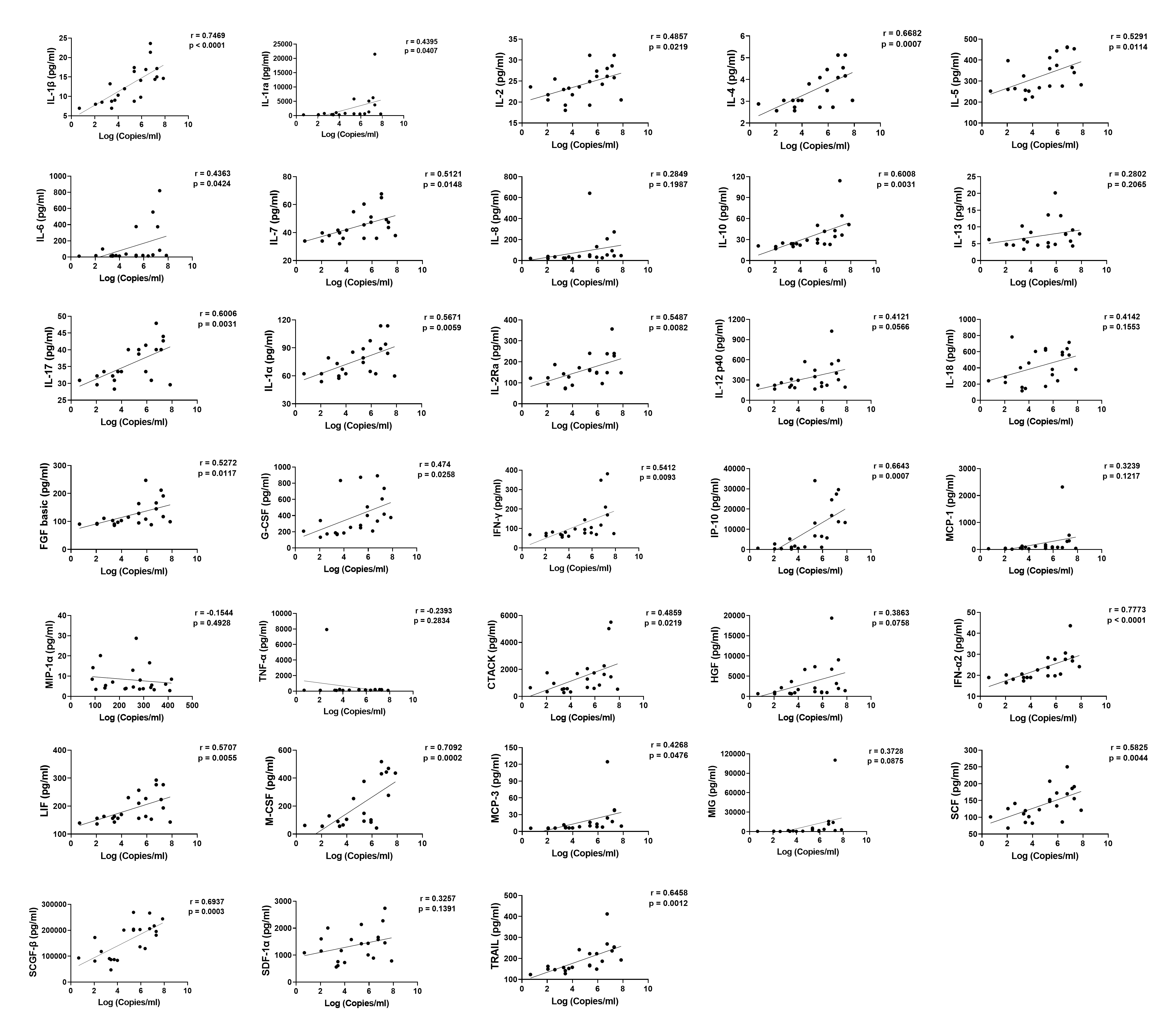

Supplement: Supplementary Figure 2 — Correlation between cytokines/chemokines expression and HAdV viral load. Expression of the 26 cytokines/chemokines showing significant increases measured from plasma samples collected upon hospital admission and thereafter and the corresponding viral load (indicated as log (copies/mL)) on the same day were evaluated using Spearman rank correlation analysis. [file Image_2.tif]

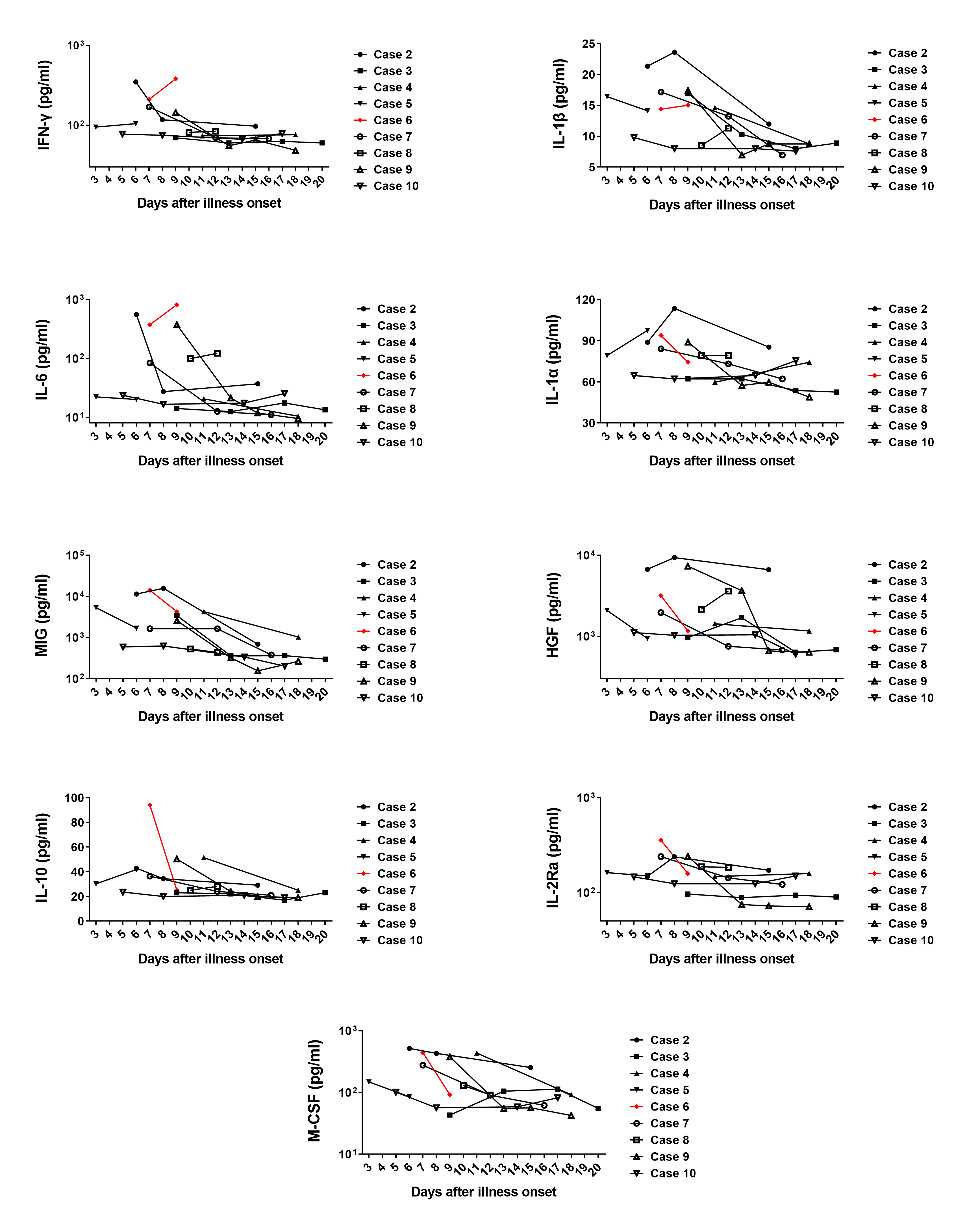

Supplement: Supplementary Figure 3 — Dynamic changes in expression of nine differentially expressed cytokines/chemokines measured in HAdV patients with or without ARDS at the indicated time-points from HAdV patients complicated with ARDS. The fatal case (case 06) is marked in red. [file Image_3.tif]

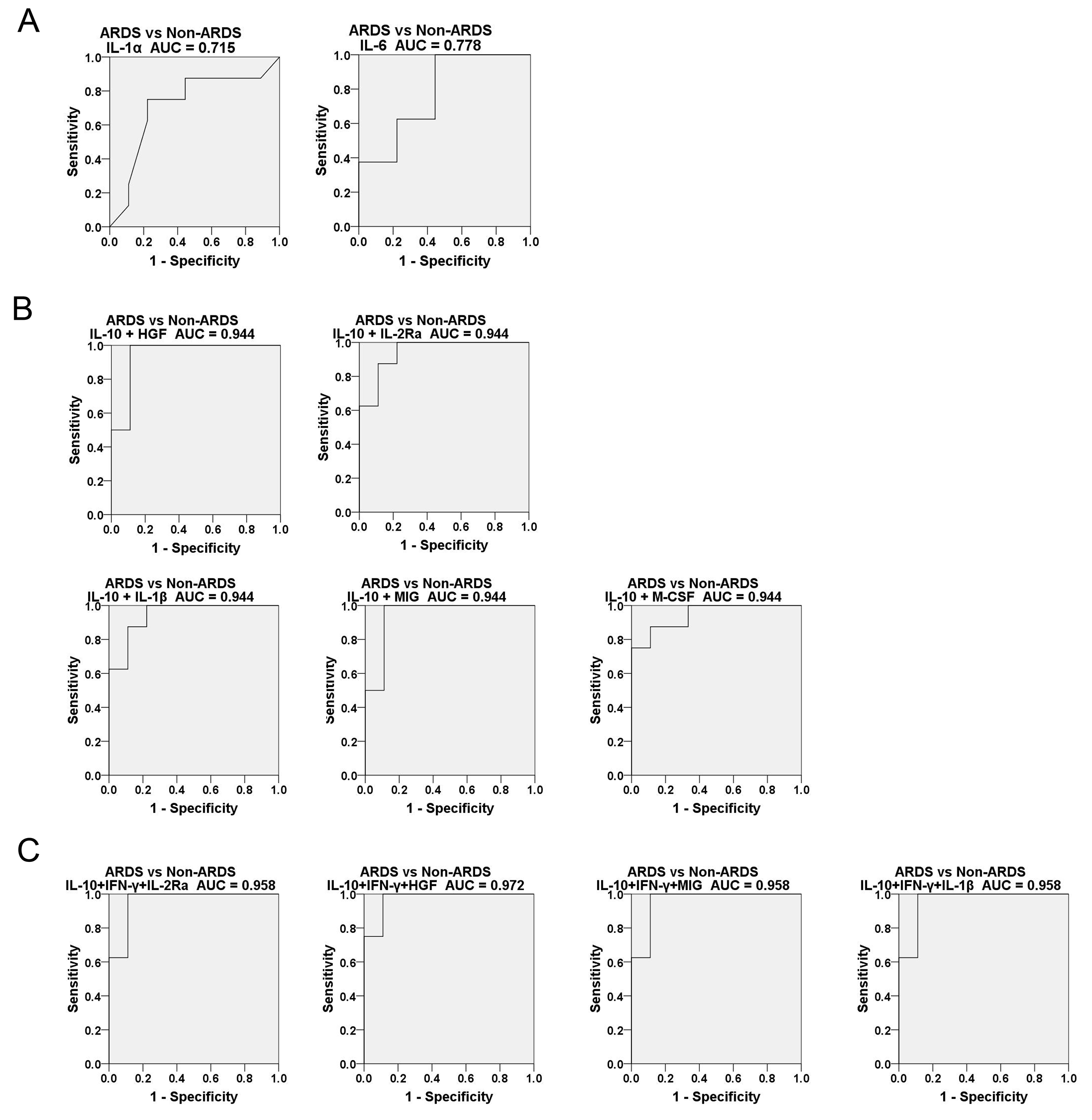

Supplement: Supplementary Figure 4 — (A) ROC curves of expression levels of IL-6 and IL-1α. (B) ROC curves of the combination of IL-10 with HGF, MIG, IL-1β, M-CSF, IL-10, and IL-2Ra expression levels upon hospital admission for HAdV-infected patients with and without ARDS during hospitalization. (C) ROC curves of the combination of IL-10, IFN-γ with HGF, MIG, IL-1β, and IL-2Ra expression levels upon hospital admission for HAdV-infected patients with and without ARDS during hospitalization. All P-values for the ROC curves were <0.05 except IL-6 and IL-1α. [file Image_4.tif]
