# Supplementary material for: Cytokine/Chemokine Expression Is Closely Associated Disease Severity of Human Adenovirus Infections in Immunocompetent Adults and Predicts Disease Progression
Source: Front Immunol. 2021 Jun 7;12:691879. doi: 10.3389/fimmu.2021.691879 (PMC8215364; doi:10.3389/fimmu.2021.691879)
Supplement: Supplementary file 5 [file Table_1.docx]

**Table S1. Epidemiological and clinical features of HAdV patients in this study.**

| **Case No.** | **Sex** | **Age** | **ARDS** | **Underling diseases** | **Initial symptoms** | **Subtypes of HAdV** | **[Virusemia](C:/Users/yylj091225/AppData/Local/youdao/dict/Application/8.9.6.0/resultui/html/index.html#/javascript:;)** | **Onset to admission (days)** | **Onset to develop ARDS (days)** | **Onset to antiviral treatment (days)** | **Antiviral agents** | **Duration of hospitalization** |
| --- | --- | --- | --- | --- | --- | --- | --- | --- | --- | --- | --- | --- |
| 01 | male | 34 | Yes | / | fever, cough, expectoration, diarrhea | HAdV-7 | NA | 5 | 5 | 9 | Oseltamivir[Ribavirin](C:/Users/yylj091225/AppData/Local/youdao/dict/Application/8.9.6.0/resultui/html/index.html#/javascript:;) Cidofovir | Transferred to other hospital |
| 02 | male | 26 | Yes | [fatty](C:/Users/yylj091225/AppData/Local/youdao/dict/Application/8.9.6.0/resultui/html/index.html#/javascript:;) [liver](C:/Users/yylj091225/AppData/Local/youdao/dict/Application/8.9.6.0/resultui/html/index.html#/javascript:;) | fever, cough | HAdV-7 | Yes | 5 | 5 | 8 | OseltamivirCidofovir | 20 |
| 03 | male | 22 | Yes | / | fever, cough, expectoration, chill | HAdV-7 | Yes | 5 | 6 | 7 | OseltamivirCidofovir | 16 |
| 04 | female | 14 | Yes | / | fever | HAdV-7 | Yes | 9 | 9 | 11 | Cidofovir | 26 |
| 05 | male | 29 | Yes | / | fever | HAdV-55 | Yes | 4 | 5 | 9 | Cidofovir | 24 |
| 06 | male | 31 | Yes | / | fever, chill | HAdV-7 | Yes | 1 | 1 | 12 | GanciclovirCidofovir | 21 |
| 07 | male | 24 | Yes | / | fever, cough, myalgia expectoration, | HAdV-55 | Yes | 13 | 14 | 17 | OseltamivirGanciclovir | 16 |
| 08 | female | 67 | Yes | / | fever, cough, expectoration, chill | HAdV-7 | Yes | 2 | 2 | 3 | OseltamivirCidofovir | Died at 9 d.a.o |
| 09 | male | 29 | Yes | / | fever, cough, headache, myalgia, expectoration, chill | HAdV-7 | Yes | 6 | 6 | 7 | [Ribavirin](C:/Users/yylj091225/AppData/Local/youdao/dict/Application/8.9.6.0/resultui/html/index.html#/javascript:;) | 17 |
| 10 | male | 49 | Yes | CHB | fever | HAdV-7 | No | 2 | 2 | 8 | [Entecavir](C:/Users/yylj091225/AppData/Local/youdao/dict/Application/8.9.6.0/resultui/html/index.html#/javascript:;) | 18 |
| 11 | male | 38 | No | / | fever | HAdV-7 | Yes | 3 | / | 6 | OseltamivirGanciclovirCidofovir | 13 |
| 12 | male | 16 | No | / | fever, cough, myalgia, expectoration, chill | HAdV-7 | Yes | 9 | / | 11 | Oseltamivir[Ribavirin](C:/Users/yylj091225/AppData/Local/youdao/dict/Application/8.9.6.0/resultui/html/index.html#/javascript:;) Cidofovir | 12 |
| 13 | male | 27 | No | / | fever, cough, headache, myalgia, expectoration, nausea or vomiting | HAdV-7 | No | 11 | / | 11 | Oseltamivir | 9 |
| 14 | female | 27 | No | / | fever, cough, headache, expectoration, chill | HAdV-7 | Yes | 1 | / | / | / | 12 |
| 15 | male | 26 | No | / | fever | HAdV-4 | Yes | 10 | / | 10 | [Ribavirin](C:/Users/yylj091225/AppData/Local/youdao/dict/Application/8.9.6.0/resultui/html/index.html#/javascript:;) Ganciclovir | 3 |
| 16 | male | 20 | No | / | fever, cough, chill | HAdV-55 | Yes | 7 | / | 10 | Oseltamivir | 25 |
| 17 | male | 38 | No | / | fever | HAdV-4 | Yes | 0 | / | / | / | 3 |
| 18 | female | 37 | No | / | fever, cough, expectoration, chill | HAdV-7 | Yes | 1 | / | 6 | Ganciclovir | 13 |
| 19 | male | 38 | No | / | fever | HAdV-4 | Yes | 1 | / | 1 | Oseltamivir | 6 |

NA: Not available.

CHB: [Chronic](C:/Users/yylj091225/AppData/Local/youdao/dict/Application/8.9.6.0/resultui/html/index.html#/javascript:;) [hepatitis](C:/Users/yylj091225/AppData/Local/youdao/dict/Application/8.9.6.0/resultui/html/index.html#/javascript:;) [B](C:/Users/yylj091225/AppData/Local/youdao/dict/Application/8.9.6.0/resultui/html/index.html#/javascript:;)

d.a.o: Days after illness onset.
