# Supplementary material for: Cytokine/Chemokine Expression Is Closely Associated Disease Severity of Human Adenovirus Infections in Immunocompetent Adults and Predicts Disease Progression
Source: Front Immunol. 2021 Jun 7;12:691879. doi: 10.3389/fimmu.2021.691879 (PMC8215364; doi:10.3389/fimmu.2021.691879)
Supplement: Supplementary file 6 [file Table_2.docx]

**Table S2. Characteristics of hospitalized patients with ARDS caused by HAdV, SARS-CoV-2, panH1N1 and bacteria in this study.**

| **Characteristics** | **HAdV**  **(N=10)** | **SARS-CoV-2**  **(N=28)** | **panH1N1**  **(N=21)** | **Bacterial**  **(N=10)** |
| --- | --- | --- | --- | --- |
| **Median age (range)** | 29 (14-67) | 63.5 (46, 74)^*^ | 53 (16, 89)^*^ | 47.5 (29-89)^*^ |
| **Age subgroups** |  |  |  |  |
| 14–59 years | 9/10 (90%) | 7/28 (25%)^*^ | 14/21 (66.7%) | 7/10 (70%) |
| ≥60 years | 1/10 (10%) | 21/28 (75%)^*^ | 7/21 (33.3%) | 3/10 (30%) |
| **Male** | 8/10 (80%) | 19/28 (67.9%) | 16/21 (76.2%) | 9/10 (90%) |
| **Initial Symptoms** |  |  |  |  |
| Fever | 10/10 (100%) | 25/28 (89.3%) | 19/21 (90.5%) | 7/10 (70%) |
| Cough | 5/10 (50%) | 19/28 (67.9%) | 20/21 (95.2%)^*^ | 5/10 (50%) |
| Headache | 1/10 (10%) | 24/28 (85.7%)^*^ | 0/21 (0%) | 1/10 (10%) |
| Myalgia | 2/10 (20%) | 17/28 (60.7%) | 0/21 (0%) | 1/10 (10%) |
| Expectoration | 5/10 (50%) | 19/28 (67.9%) | 9/21 (42.9%) | 4/10 (40%) |
| Chill | 5/10 (50%) | 4/28 (14.3%)^*^ | 1/21 (4.8%)^*^ | 2/10 (20%) |
| Nausea or vomiting | 0/10 (0%) | 0/28 (0%) | 3/21 (14.3%) | 1/10 (10%) |
| Diarrhea | 1/10 (10%) | 3/28 (10.7%) | 0/21 (0%) | 0/10 (0%) |
| **Tmax, ℃§** | 40 (39.5-40.4) | 38.8 (38.38-39.1) | 39 (38.6-39.2) | 39.3 (38.93, 39.6) |
| **Underlying diseases** | 2/10 (20%) | 18/28 (64.3%)^*^ | 20/21 (95.2%)^*^ | 6/10 (60%) |
| Chronic heart disease | 0/10 (0%) | 14/28 (50%)^*^ | 7/21 (33.3%) | 2/10 (20%) |
| Chronic lung disease | 0/10 (0%) | 0/28 (0%) | 7/21 (33.3%) | 1/10 (10%) |
| Chronic renal disease | 0/10 (0%) | 0/28 (0%) | 1/21 (4.8%) | 0/10 (0%) |
| Chronic liver disease | 2/10 (20%) | 2/28 (7.2%) | 0/21 (0%) | 1/10 (10%) |
| Diabetes | 0/10 (0%) | 2/28 (7.2%) | 3/21 (14.3%) | 0/10 (0%) |
| Cancer | 0/10 (0%) | 1/28 (3.6%) | 0/21 (0%) | 0/10 (0%) |
| **Bacterial co-infections** | 3/10 (30%) | 7/28 (25%) | 18/21 (85.7%)^*^ | NA |
| **Complications** |  |  |  |  |
| Pneumonia | 10/10 (100%) | 28/28 (100%) | 21/21 (100%) | 10/10 (100%) |
| ARDS | 10/10 (100%) | 28/28 (100%) | 21/21 (100%) | 10/10 (100%) |
| Severe ARDS | 4/10 (40%) | 4/28 (14.3%) | 10/21 (47.6%) | 2/10 (20%) |
| Respiratory failure | 7/10 (70%) | 28/28 (100%)^*^ | 17/21 (80.9%) | 8/10 (80%) |
| Hepatic insufficiency | 7/10 (70%) | 10/28 (35.7%) | 4/21 (19%)^*^ | 2/10 (20%) |
| Renal insufficiency | 3/10 (30%) | 7/28 (25%) | 6/21 (28.6%) | 5/10 (50%) |
| Cardiac failure | 3/10 (30%) | 2/28 (7.2%) | 1/21 (4.8%) | 5/10 (50%) |
| Shock | 2/10 (20%) | 1/28 (3.6%) | 4/21 (19%) | 6/10 (60%) |
| Viremia | 9/10 (90%) | 0/28 (0%)^*^ | 0/21 (0%)^*^ | NA |
| **Treatment** |  |  |  |  |
| Antiviral | 10/10 (100%)† | 28/28 (100%) | 21/21 (100%) | NA |
| Cidofovir | 7/10 (70%) | NA | NA | NA |
| Corticosteroid | 8/10 (80%) | 10/28 (35.7%)^*^ | 9/21 (42.9%) | 5/10 (50%) |
| Mechanical ventilation | 9/10 (90%) | 21/28 (75%) | 16/21 (76.2%) | 9/10 (90%) |
| Invasive mechanical ventilation | 4/9 (44.4%) | 7/28 (25%) | 7/21 (33.3%) | 4/10 (40%) |
| Immunoglobulin | 4/10 (40%) | 12/28 (42.9%) | 3/21 (14.3%) | 0/10 (0%)^*^ |
| Intentsive care unit (ICU) | 9/10 (90%) | 20/28 (71.4%) | 16/21 (76.2%) | 9/10 (90%) |
| **Interval, median days (IQR)^+^** |  |  |  |  |
| Onset to admission | 5 (2.5, 6.5) | 4 (2, 5) | 5 (3, 7) | 2 (1.25, 3) ^*^ |
| Onset to starting antiviral treatment | 8.5 (7.25, 10.5) | 4 (3.75, 6.25) ^*^ | 5 (4, 7) ^*^ | NA |
| Onset to laboratory confirmation | 8 (7, 9) | 4 (3, 6)^*^ | 5 (4, 8) | 5.5 (3.5, 7,75) |
| **Case fatality rate** | 1/10 (10%) | 1/28 (3.6%) | 2/21 (9.5%) | 5/10 (50%) |
| **Hospitalization (days, IQR)** | 19 (16.75, 21.75) | 23 (18.25, 37.75) | 21 (10, 21) | 22 (17, 31) |

^+^IQR, inter-quartile range

† Treatment with [Ribavirin](C:/Users/Administrator/AppData/Local/youdao/dict/Application/8.5.3.0/resultui/html/index.html#/javascript:;), [Ganciclovir](C:/Users/Administrator/AppData/Local/youdao/dict/Application/8.5.3.0/resultui/html/index.html#/javascript:;) or Cidofovir.

§ Median (IQR).

NA: Not available.

*Statistically significant with HAdV group.
